# Occurrence of KPC-Producing *Escherichia coli* in Psittaciformes Rescued from Trafficking in Paraíba, Brazil

**DOI:** 10.3390/ijerph18010095

**Published:** 2020-12-25

**Authors:** Gedean Galdino da Cruz Silva, Eloiza Helena Campana, Priscylla Carvalho Vasconcelos, Núbia Michelle Vieira da Silva, Lauro Santos Filho, Elma Lima Leite, Patrícia Emília Naves Givisiez, Wondwossen Abebe Gebreyes, Celso José Bruno de Oliveira

**Affiliations:** 1 Laboratório de Avaliação de Produtos de Origem Animal (LAPOA), Departamento de Zootecnia, Centro de Ciências Agrárias, Universidade Federal da Paraíba, Areia-PB 58397-000, Brazil; gedean_gcs@hotmail.com (G.G.d.C.S.); pczootecnista.1@gmail.com (P.C.V.); nubia274@gmail.com (N.M.V.d.S.); limaleiteelma@gmail.com (E.L.L.); patricia@cca.ufpb.br (P.E.N.G.); 2 Laboratório de Microbiologia Clínica (LMC), Departamento de Ciências Farmacêuticas, Centro de Ciências da Saúde, Universidade Federal da Paraíba, João Pessoa-PB 58033-455, Brazil; elocampana@ccs.ufpb.br (E.H.C.); lauro.ufpb@hotmail.com (L.S.F.); 3 Global One Health Initiative (GOHi), Ohio State University, Columbus, OH 43210, USA; gebreyes.1@osu.edu

**Keywords:** antimicrobials, resistance genes, wildlife

## Abstract

The emergence and spread of antimicrobial resistance pose a threat to public health globally. Antibiotic-resistant bacteria and genes can disseminate among environments, animals and humans. Therefore, investigation into potential reservoirs of multidrug-resistant bacteria is of great importance to the understanding of putative transmission routes of resistant bacteria and resistance genes. This study aimed to report the occurrence of *Escherichia coli* harboring the *Klebsiella pneumoniae* carbapenemase-producing gene (*bla*_KPC_) in Psittaciformes rescued from wildlife trafficking in Paraíba State, Brazil. Cloacal swabs were collected from thirty birds and cultured by conventional microbiology using MacConkey and serum tryptone glucose glycerol (STGG) media supplemented with selective antimicrobials. *E. coli* isolates (n = 43) were identified by phenotypic tests and confirmed by MALDI-TOF. Antimicrobial susceptibility profiles were determined by means of Kirby–Bauer test. All isolates were further screened for extended-spectrum beta-lactamase (ESBL) production, and putative genes encoding ESBL were investigated by PCR. Additionally, *bla*_KPC_-harboring strains were genotyped by REP-PCR. A total of 43 *E. coli* phenotypically resistant isolates were recovered. The highest resistance rate was observed against ciprofloxacin. Among the resistance genes, only *bla*_KPC_ was found in seven different birds from three species. According to the genotyping, these seven isolates belonged to four different strains. To date, this is the first report on the occurrence of KPC-*E. coli* in Psittaciformes rescued from trafficking in Northeastern Brazil. Due to the high clinical importance of KPC-*E. coli*, our findings suggest that wild animals in captivity at wildlife rescue centers can play a role as reservoirs of bacteria that are resistance to Critically Important antimicrobials in human medicine.

## 1. Introduction

Antimicrobial resistance is a major threat to global public health. Despite being a natural phenomenon, the emergence and rapid dissemination of resistant bacteria are directly related to the inadequate use of antimicrobials, the unavailability of new drugs, the incorrect disposal of drugs and the inadequate treatment of effluents, generating serious clinical and economic consequences associated with increased morbidity and mortality of patients [1]. Therefore, antimicrobial resistance must be understood as a One Health issue involving epidemiological aspects related to humans, animals and the environment [2].

Although a large number of studies on antimicrobial resistance have been conducted and reported, most of them relate to humans and companion animal species. There is a lack of information on the potential role of wildlife in the epidemiology of antimicrobial resistance. Theoretically, wild birds could play a relevant role, as they inhabit many ecological niches and act as biomarkers, being able to acquire and disseminate antimicrobial-resistant microorganisms from human, livestock, or environmental sources. Antimicrobial-resistant *E. coli* have been identified among various European wild bird species, potentially serving as reservoirs of antimicrobial resistance genes [3]. There is evidence that some wild bird species can acquire antimicrobial-resistant strains directly from individuals during migration or through exposure to residues [4], even though antimicrobial resistance in wildlife seems to be more complex than simple anthropogenic causes [5].

The potential problems associated with antimicrobial resistance in wildlife are still poorly comprehended. In conservation captivity for instance, drug screening may be compromised and the reintroduction of rehabilitated species may be disrupted, as these animals could be reservoirs of multiresistant microorganisms to other free-living, domestic breeding species and humans [6]. The increasing interest in birds as pets is also a concern. Psittaciformes are one of the most common pets due to their easy adaptation, plumage colors and ability to imitate human sounds [7]. Bacterial resistance to critically important antimicrobials, such as carbapemens, which is normally associated with additional resistance against drugs of other classes [8], poses a risk to individuals in close contact with those birds.

The aim of this study was to report *Klebsiella pneumoniae* carbapenemase-producing - *Escherichia coli* (KPC *E. coli*) in Psittaciformes rescued from wildlife trafficking in Paraíba State, Brazil.

## 2. Materials and Methods

### 2.1. Study Design and Sampling

The study was performed at the Center for Rescued Wild Animals of Paraíba State (*Centro de Triagem de Animais Silvestres da Paraíba* (CETAS-PB)), located in the *Restinga de Cabedelo* National Forest (7°03′46.9″ S, 34°51′22.0″ O), under the approval of the Biodiversity Authorization and Information System no. 65316-1. Animal handling and sampling procedures were previously approved by the Ethics Committee on Animal Use of the Federal University of Paraíba (CEUA N° 9504051018).

Duplicate cloacal swabs were collected from thirty newly rescued or rehabilitated Psittaciformes birds (Table 1). The species were captured from their enclosures with the aid of a polypropylene mesh and manually contained for later identification, clinical evaluation and collection of biological samples. Two sterile urethral swabs were used to sample the cloacal microbiota and individually placed into 4 mL of MacConkey broth (Kasvi, Brazil) and serum tryptone glucose glycerol (STGG) broth. The latter was prepared in-house using skim milk, tryptone, glucose and glycerin, as previously described [9]. The samples were stored in a thermal box and transported to the Clinical Microbiology Laboratory of the Federal University of Paraíba.

### 2.2. Microbial Isolation

STGG samples were processed within two hours of sampling, and MacConkey broth samples were incubated at 37 °C for 24 h. For both media, 100 µL-aliquots were transferred to three MacConkey agar plates (BD, Franklin Lakes, NJ, USA), each supplemented with the following selective antimicrobials: (I) ceftriaxone 8 µg/mL; (II) imipenem 1 µg/mL; and (III) polymyxin B 3.5 µg/mL. Plates were incubated aerobically at 35 ± 2 °C for 18–24 h. The morphological characteristics of the colonies were reported, and representative colonies were later transferred to tryptic soy agar (TSA) medium (BD), also supplemented with the same antimicrobial used in the original MacConkey plate. All recovered strains were stored at −80 °C in sterile cryopreservation tubes containing skim milk solution, distilled water and 15% glycerin.

### 2.3. Bacterial Identification

The isolates were phenotypically identified by biochemical tests in solid (Simmons Citrate, Triple Sugar Iron agar—TSI, Lysine Iron Agar—LIA, Urea and Phenylalanine) and semisolid media (Motidade, Indol and Ornithina—MIO).

Confirmation of bacterial species was performed by means of mass spectrometry—flight time (MALDI-TOF MS) using the Bruker BioTyper (Bruker Corporation, Billerica, MA, USA). Briefly, the isolates on TSA agar were inoculated in triplicate directly into a 96-spot steel microplate (Bruker Daltonics). After air drying, 1 µL of matrix solution (α-cyano-4-hydroxycinnamic acid) was placed over each inoculum. Raw spectra were processed and compared to a reference database using MALDI BioTyper software version 3.1 (Bruker Daltonics). The following standard Bruker interpretative criteria were used: unreliable identification (score 0.000–1.699); probable genus identification (score 1.700–1.999); secure genus and probable species identification (score 2.000–2.299); highly probable species identification (score 2.300–3.000). Therefore, reliable species identification was considered when a minimum 2.3 score was obtained in at least two wells for a given isolate.

### 2.4. Antimicrobial Susceptibility Testing and ESBL Screening

Antimicrobial susceptibility testing was performed by disk diffusion [10] for sulfamethoxazole + trimethoprim (25MCG); ertapenem (10MCG); meropenem (10MCG); imipenem (10MCG); amikacin (30MCG); ciprofloxacin (5MCG); tetracycline (30MCG); chloramphenicol (30MCG); ceftriaxone (30MCG); ceftazidime (30MCG); ampicillin + sulbactam (20MCG); aztreonam (30MCG) and amoxicillin/clavulanic-acid (30MCG). *E. coli* ATCC 25,922 and *Pseudomonas aeruginosa* ATCC 27,853 were used as quality controls, and results were interpreted according to the Clinical Laboratory Standard Institute [11]. ESBL detection was carried out by double-disk synergy test (DDS), as previously described [12].

### 2.5. Detection of Genes Encoding for Resistance by PCR and Genetic Relatedness Analysis by REP-PCR

Genomic DNA was extracted by the modified phenol-chloroform-isoamyl alcohol method [13] and used as a template in the PCR assays targeting the genes *bla*_CTX_, *bla*_NDM_, *bla*_KPC_ and *mcr*-1. The primers and cycling conditions are shown in Table 2. KPC-positive *E. coli* isolates were genotyped by Repetitive Extragenic Palindromic Sequences PCR (REP-PCR), as previously described [14].

## 3. Results

Out of the thirty sampled birds, 19 (63.3%) showed bacterial growth in at least one of the three antimicrobial-supplemented agar dishes. A higher recovery was observed in MacConkey broth (28/90; 31.1%) in comparison with STGG (13/90; 14.4%). This difference might be associated with the longer incubation period (24–48 h) for samples in MacConkey broth. Considering the three types of antimicrobial supplementation, a higher frequency was observed in the plates containing polymyxin B (Table 3).

The antimicrobial susceptibility profiles of the *E. coli* isolates are shown in Table 4. The highest resistance rates were observed for ciprofloxacin (11.7%), ertapenem (9.3%) and ceftazidime (9.3%). The majority of the isolates were susceptible to different classes of antimicrobials, such as aztreonam (95.2%), sulfamethoxazole + trimethoprim (93.0%), imipenem (93.0%), amikacin (93.0%), tetracycline (93.0%), chloramphenicol (93.0%) and ceftriaxone (93.0%). In addition, no ESBL phenotype was detected among the investigated isolates.

Among the antimicrobial resistance genes targeted by PCR, *bla*_KPC_ was the only resistance determinant detected in 13 isolates originated from seven distinct bird species: one orange-winged parrot (*Amazona amazonica*), one scarlet macaw (*Ara macao*), one red-and-green macaw (*Ara chloropterus*), one blue-and-yellow macaw (*Ara ararauna*), two turquoise-fronted parrots (*Amazona aestiva*) and one festive parrot (*Amazona festiva*) (Table 5).

**Figure 1:**
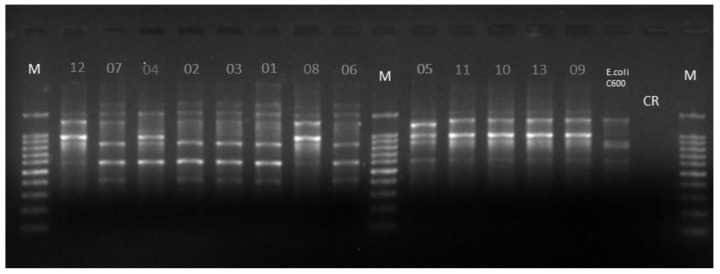
Genotypic relatedness of *Klebsiella pneumoniae* carbapenemase-producing - *Escherichia coli* (KPC *E. coli*) isolated from trafficked Psittaciformes under captivity at the Centro de Triagem de Animais Silvestres da Paraíba (CETAS-PB), located in the Restinga de Cabedelo National Forest, Brazil.Genotype I: samples 08, 09, 10, 11, 12 and 13; Genotype II: samples 01, 02, 03, 06 and 07; Genotype III: sample 04 and Genotype IV: sample 05.

The genotypic relatedness analysis by REP-PCR (Figure 1) indicated that the 13 *bla*KPC-harboring *E. coli* clustered in four distinct genotypes (genotype I: samples 08, 09, 10, 11, 12 and 13 from three *Amazona* species; genotype II: samples 01, 02, 03, 06 and 07 from two *Ara* species; genotype III: sample 04 from *Ara macao*; and genotype IV: sample 05 from *Ara chloropterus*) (Figure 1). It is worth noting that two different KPC-*E. coli* strains (No. 05/genotype IV and No. 06/genotype II) were recovered from a single *Ara chloropterus* bird (ARA 5). Interestingly, genotype I predominated among *E. coli* recovered from *Amazona* birds, while genotypes II and IV were detected in the genus *Ara* only.

Only strains 09 and 13 (genotype I) were recovered from MacConkey media supplemented with imipenem, whereas the majority of the *bla*KPC-*E. coli* were isolated from STGG media supplemented with polymyxin B.

## 4. Discussion

The detection of *bla*_KPC_ in 13 different *E. coli* isolates harboring no *bla*_CTX-M_ gene could explain their observed phenotypic antimicrobial resistance patterns. However, these isolates have not been investigated for other extended-spectrum beta-lactamase genes that are commonly found in *E. coli*, such as *bla*_TEM_ and *bla*_SHV_. Although there was a higher isolation rate of antimicrobial-resistant bacteria in MacConkey compared with STGG broth, the majority of the *bla*KPC-*E. coli* were recovered from STGG media supplemented with polymyxin B. This interesting finding indicates that imipenem-resistant *E. coli* are probably more demanding in terms of bacteriological cultivation, and highlights the potential limitations of protocols targeting the detection of antimicrobial-resistant bacteria.

Human activities such as degradation and fragmentation of natural habitats force interaction among humans, wildlife and domestic animals, favoring the transmission of multiresistant bacteria among different species. *E. coli* are commensal bacteria abundantly present in the intestinal microbiota of animal species. In this context, the spread of antimicrobial resistance among abundant species such as *E. coli* poses a concern, since acquired resistance in these bacteria is frequently associated with mobile genetic elements, such as plasmids, transferred by horizontal mechanisms [5]. On the other hand, anthropogenic causes have not been clearly identified as major drivers determining antimicrobial resistance in wildlife [6].

Little is known about the true protocooperative role of *E. coli* in the enteric functioning of the hosts. It is known that, in wild birds, some pathogenic strains can lead to severe clinical conditions [18]. According to Sanches [19], birds are commonly infected with enteropathogenic *Escherichia coli* (EPEC) strains, a typical pathotype for these animals. Previous studies have reported greater intestinal colonization by EPEC in captive parrots compared to free-living birds [18]. Such findings can possibly be explained by factors related to feeding, hygiene of the enclosures and the use of antimicrobials. Under captivity, Psittaciformes may play an important role in maintaining cycles of antimicrobial-resistant enteric colibacillosis, with a potential impact on public health.

The transmission of resistant bacterial strains in wild species is directly linked to contact with effluents, elimination of solid residues, excreta of farm animals and propagation by synanthropic rodents [20]. Another important driver is migratory birds, which may carry bacteria harboring resistance determinants of emerging importance [15]. As reported by Dolejska and Literak [21], *bla*_KPC-2_ and *bla*_OXA-48_ genes were reported in *E. coli* from seagulls. Low but frequent detection of carbapenemase-producing *E. coli* in wild birds in Alaska may indicate environmental dissemination of these bacteria in sites with relatively low levels of food animal farming and without previous evidence of carriage by the human clinical population [22].

The *bla*_KPC_ gene has already been reported among *E. coli* from different bird species belonging to the Columbiformes, Passeriformes, Charadriiformes, Accipitriformes, Falconiformes and Strigiformes orders [23]. In Brazil, Pontes et al. [24] detected the resistance genes *str*AB, *bla*TEM, *tet*A, *tet*B, *aad*A, *apha*A, *sul*1, *sul*2, and *sul*3 associated with plasmids in cloacal avian pathogenic *Escherichia coli* (APEC) strains from *Nymphicus hollandicus* in São Paulo and Rio de Janeiro. Cloacal *E. coli* from *Pyrrhura griseipectus* was phenotypically resistant to azithromycin, sulfamethoxazole-trimethoprim, streptomycin and tetracycline [25]. Resistance to tobramycin and streptomycin was observed in cloacal *E. coli* from *Amazona aestiva*, *Ara chloroptera* and *Ara ararauna* from Parque das Aves de Toledo, Paraná (Brazil) [7]. However, surveillance of antimicrobial-resistant bacterial strains in wildlife is usually performed by means of phenotypic testing.

Besides the high number of *E. coli* strains carrying *bla*_KPC_, the fact that two unrelated KPC-*E. coli* strains were recovered from a single bird (ARA 5) suggests that this gene could be disseminated through mobile genetic elements such as plasmids.

The first carbapenemase-producing *E. coli* in wild birds was associated with *Milvus migrans* in Germany [26]. Our study is the first to report Psittaciformes as carriers of carbapenemase-producing *E. coli* in Brazil. Considering most studies in wildlife do not target molecular mechanisms of antimicrobial resistance and normally report phenotypic resistance patterns of bacteria, our results suggest that Psittaciforms are still underestimated as potential reservoirs of multiresistant Enterobacteriacea. Considering the increasing importance of Psittaciforms as pets and their high abundance in rescue centers, further studies are warranted to understand the role of these animals in the epidemiology of antimicrobial resistance and the public heath impact.

## 5. Conclusions

The occurrence of KPC-producing *E. coli* in the cloaca of Psittaciformes rescued from trafficking highlights the potential role of wildlife birds in the epidemiology of antimicrobial resistance. In view of the increasing importance of rescue and rehabilitation centers around the world, attention is needed in order to avoid hard-to-treat occupational infections in humans in close contact to the birds, and also mitigate the dissemination of antimicrobial resistance associated with the return of birds to native environments.

## Figures and Tables

**Table 1:** Psittaciformes species sampled for cloacal swabs at the Centro de Triagem de Animais Silvestres da Paraíba (CETAS-PB), located in the Restinga de Cabedelo National Forest, Paraíba State, Brazil.

Species	Popular Name	Number of Individuals
*Amazona aestiva*	Turquoise-fronted parrot	10
*Amazona amazonica*	Orange-winged parrot	2
*Amazona festiva*	Festive parrot	1
*Ara ararauna*	Blue-and-yellow macaw	3
*Ara chloropterus*	Red-and-green macaw	3
*Ara macaw*	Scarlet macaw	1
*Diopsittaca nobilis*	Red-shouldered macaw	5
*Eupsittula aurea*	Peach-fronted parakeet	1
*Eupsittula cactorum*	Cactus parakeet	1
*Forpus xanthopterygius*	Blue-winged parrotlet	1
*Psittacara leucophthalmus*	White-eyed parakeet	1
*Thectocercus acuticaudatus*	Blue-crowned parakeet	1
		**Total**: 30

**Table 2:** Oligonucleotide primers and thermal cycling conditions used in the PCR assays targeting genes conferring resistance against beta-lactamase (CTX-M), carbapenems (NDM and KPC) and colistin (MCR-1).

Genes Encoding for Beta-Lactamase Resistance				
*Gene*	*Sequences (5-3′)*	*Amplicon size* (*BP* ^1^)	*TC* ^2^	*Reference*
*bla*CTX-M F	SCSATGTGCAGYACCAGTAA	554	1	[15]
*bla*CTX-M R	CCGCRATATGRTTGGTGGTG			
**Genes Encoding for Carbapenems Resistance**				
*Gene*	*Sequences (5-3′)*	*Amplicon* (*BP* ^1^)	*TC* ^2^	*Reference*
*bla*NDM F	GGTTTGGCGATCTGGTTTTC	621	2	[16]
*bla*NDM R	CGGAATGGCTCATCACGATC			
*bla*KPC-2 F	TCGCCGTCTAGTTCTGCTGTCTTG	800	3	[17]
*bla*KPC-3 R	CAATCCCTCGAGCGCGAGTC			
**Genes Encoding for Colistin Resistance**				
*Gene*	*Sequences (5-3′)*	*Amplicon* (*BP* ^1^)	*TC* ^2^	*References*
MCR-1 F	GATCGGATTGGAGAACCAGA	343	4	[15]
MCR-1 R	ATTTCTGACCGCATTTCCAT			

^1^ BP: base pairs. ^2^ TC: thermocycling conditions. 1 = 94 °C 3 min; 35 cycles of 94 °C 30 s, 55 °C 30 s, 72 °C 45 s; e 72 °C 5 min. 2 = 94 °C 10 min; 36 cycles of 94 °C 30 s, 52 °C 40 s, 72 °C 50 s; e 72 °C 5 min. 3 = 95 °C 7 min; 35 cycles of 95 °C 40 s, 54 °C 45 s, 72 °C 40 s, e 72 °C 10 min. 4 = 94 °C 15 min; 25 cycles of 94 °C 30 s, 59 °C 90 s, 72 °C 60 s; 72 °C 10 min.

**Table 3:** Positive cloacal swabs and number of *E. coli*-confirmed samples from Psittaciformes under captivity at the Centro de Triagem de Animais Silvestres da Paraíba (CETAS-PB), located in the Restinga de Cabedelo National Forest, Brazil.

Broth	Growth	Antimicrobial Supplementation			Number of Isolates	Confirmed *E. coli* -Positive Isolates
		*Cef* ^1^	*Imi* ^2^	*Poly B* ^3^		
MacConkey	28/90	05/28	7/28	16/28	36/71	25/71
STGG ^4^	13/90	03/13	0/13	10/13	24/71	19/71

^1^*Cef*: ceftriaxone; ^2^*Imi*; imipenem; ^3^*Poly B*: polymyxin B; ^4^ STGG: skim milk, tryptone, glucose and glycerin.

**Table 4:** Susceptibility profiles of cloacal *E. coli* isolated from trafficked Psittaciformes under captivity at the Centro de Triagem de Animais Silvestres da Paraíba (CETAS-PB), located in the Restinga de Cabedelo National Forest, Brazil.

Antimicrobials	Initials	Susceptibility Profile					
		S ^1^	%	I ^2^	%	R ^3^	%
Sulfamethoxazole + trimethoprim _(25MCG)_	*SUT*	40	93.0	1	2.4	2	4.6
Ertapenem _(10MCG)_	*ETP*	36	83.7	3	7.0	4	9.3
Meropenem _(10MCG)_	*MER*	38	88.4	3	7.0	2	4.6
Imipenem _(10MCG)_	*IPM*	40	93.0	3	7.0	0	0.0
Amikacin _(30MCG)_	*AMI*	40	93.0	3	7.0	0	0.0
Ciprofloxacin _(5MCG)_	*CIP*	31	72.0	7	16.3	5	11.7
Tetracycline _(30MCG)_	*TET*	40	93.0	1	2.4	2	4.6
Chloramphenicol _(30MCG)_	*CLO*	40	93.0	1	2.4	2	4.6
Ceftriaxone _(30MCG)_	*CRO*	40	93.0	2	4.6	1	2.4
Ceftazidime _(30MCG)_	*CAZ*	37	86.1	2	4.6	4	9.3
Ampicillin + sulbactam _(20MCG)_	*APS*	38	88.4	2	4.6	3	7.0
Aztreonam _(30MCG)_	*ATM*	41	95.2	1	2.4	1	2.4
Amoxicillin/acid-clavulanic _(30MCG)_	*ACM*	39	90.6	1	2.4	3	7.0

^1^ S: susceptible; ^2^ I: intermediate; ^3^ R: resistant.

**Table 5:** *bla*KPC-positive *E. coli* isolated from trafficked Psittaciformes kept at the Centro de Triagem de Animais Silvestres da Paraíba (CETAS-PB), located in the Restinga de Cabedelo National Forest, Brazil.

Sample Number	Species	Animal Id ^1^	Enrichment Broth ^2^	ATB Supplementation ^3^	Genotype ^4^
01	*Ara ararauna*	ARA 2	STGG	Poli B	II
02	*Ara ararauna*	ARA 2	STGG	Poli B	II
03	*Ara ararauna*	ARA 2	STGG	Poli B	II
04	*Ara macao*	ARA 4	STGG	Poli B	III
05	*Ara chloropterus*	ARA 5	STGG	Poli B	IV
06	*Ara chloropterus*	ARA 5	STGG	Poli B	II
07	*Ara chloropterus*	ARA 5	STGG	Poli B	II
08	*Amazona aestiva*	PAP 8	STGG	Poli B	I
09	*Amazona aestiva*	PAP 8	MAC	Imi	I
10	*Amazona festiva*	PAP 9	STGG	Poli B	I
11	*Amazona amazonica*	PAP 11	STGG	Poli B	I
12	*Amazona amazonica*	PAP 11	STGG	Poli B	I
13	*Amazona aestiva*	PAP 12	MAC	Imi	I

^1^ Animal identification: ARA (*Ara genus*) or *Amazona genus* (PAP); ^2^ Enrichment broth showing bacterial growth: STGG (skim milk, tryptone, glucose and glycerin) or MacConkey (MAC); ^3^ Type of antibiotic supplementation in enrichment broth showing bacterial growth: polymyxin B (PoliB) or imipenem (IMI); ^4^ Genotypic pattern by means of Rep-PCR.
